# Task-relevant brain networks identified with simultaneous PET/MR imaging of metabolism and connectivity

**DOI:** 10.1007/s00429-017-1558-0

**Published:** 2017-11-13

**Authors:** Andreas Hahn, Gregor Gryglewski, Lukas Nics, Lucas Rischka, Sebastian Ganger, Helen Sigurdardottir, Chrysoula Vraka, Leo Silberbauer, Thomas Vanicek, Alexander Kautzky, Wolfgang Wadsak, Markus Mitterhauser, Markus Hartenbach, Marcus Hacker, Siegfried Kasper, Rupert Lanzenberger

**Affiliations:** 1 Department of Psychiatry and Psychotherapy, Medical University of Vienna, Waehringer Guertel 18-20, 1090 Vienna, Austria; 2 Division of Nuclear Medicine, Department of Biomedical Imaging and Image-guided Therapy, Medical University of Vienna, Vienna, Austria; 3 Center for Biomarker Research in Medicine (CBmed), Graz, Austria; 4 Ludwig Bolzmann Institute Applied Diagnostics, Vienna, Austria

**Keywords:** Continuous task performance, Functional connectivity, Glucose metabolism, White matter microstructure, Functional PET, fPET

## Abstract

Except for task-specific functional MRI, the vast majority of imaging studies assessed human brain function at resting conditions. However, tracking task-specific neuronal activity yields important insight how the brain responds to stimulation. We specifically investigated changes in glucose metabolism, functional connectivity and white matter microstructure during task performance using several recent methodological advancements. Opening the eyes and right finger tapping had elicited an increased glucose metabolism in primary visual and motor cortices, respectively. Furthermore, a decreased metabolism was observed in the regions of the default mode network, which allowed absolute quantification of commonly described deactivations during cognitive tasks. These brain regions showed widespread task-specific changes in functional connectivity, which stretched beyond their primary resting-state networks and presumably reflected the level of recruitment of certain brain regions for each task. Finally, the corresponding white matter fiber pathways exhibited changes in axial and radial diffusivity during the tasks, which were regionally distinctive for certain tract groups. These results highlight that even simple task performance leads to substantial changes of entire brain networks. Exploiting the complementary nature of the different imaging modalities may reveal novel insights how the brain processes external stimuli and which networks are involved in certain tasks.

**Electronic supplementary material:**

The online version of this article (10.1007/s00429-017-1558-0) contains supplementary material, which is available to authorized users.

## Introduction

Non-invasive neuroimaging techniques provide us with the opportunity to characterize the human brain at multiple levels of structure and function. Among others, this includes microstructural properties of gray (Sagi et al. 2012) and white matter (Giorgio et al. 2010), oxygen demand (Logothetis 2008) and glucose consumption (Stender et al. 2015) as metrics of neuronal activation and connectivity across brain regions (Fox and Raichle 2007; Biswal et al. 2010). Except for functional magnetic resonance imaging (fMRI), the vast majority of studies assessed such imaging parameters at rest, mostly comparing patient cohorts to healthy controls. Although these investigations have demonstrated fundamental insight into the human brain and alterations thereof, task-relevant image acquisition yields specific information how the brain processes and responds to external stimulation, which in turn can be directly linked to performance and behavior. Studies focusing on task-relevant changes in glucose metabolism and connectivity are still scarce and were mostly carried out using a single imaging modality. Using positron emission tomography (PET), task-specific changes in glucose metabolism had to be assessed using repeated measurements (Phelps et al. 1981; Cahill et al. 1996) and injections (Nishizawa et al. 2001) or even different groups (Riedl et al. 2014), leading to high intra- and intersubject variability of ~ 25% (Schmidt et al. 1996). On the other hand, task-relevant functional connectivity has often been computed from conventional fMRI approaches (Honey et al. 2002; Fair et al. 2007), despite the fact that these were actually designed to study neuronal activation in response to 10–20 s stimulation instead of connectivity across several minutes. Hence, such approaches include disadvantages in terms of high-frequency signal changes between rest and task, which may in turn cause pronounced differences in connectivity as compared to continuous acquisitions (Ganger et al. 2015).

Here, we employed several methodological advancements to specifically study task-relevant activation in the human brain. First, the use of a hybrid PET/MR system decreases intrasubject variability since several imaging modalities are acquired simultaneously. This avoids repeated measurements on separate imaging systems as well as the accompanied attentional bias and adaptation, which would in turn directly affect task performance and hence brain activation (Sladky et al. 2012; Hahn et al. 2013). Second, we applied a novel approach for task-specific quantification of glucose metabolism, which enables evaluation of several task conditions in a single measurement (Hahn et al. 2016a). Finally, changes in functional connectivity and white matter microstructure were assessed for tasks that were continuously executed during the acquisition (Shirer et al. 2012). Combining the above techniques we aimed to identify brain networks involved in the performance of simple but well-established tasks in a multimodal fashion.

## Materials and methods

### Subjects

Eighteen right-handed healthy subjects were included in this study (24.2 ± 4.3 years, 8 females). Fifteen of them already participated in a previous study (Hahn et al. 2016a). At the screening visit, all subjects were assessed by an experienced psychiatrist to rule out psychiatric disorders as well as neurological or somatic diseases. This included the Structural Clinical Interview for DSM-IV and a routine medical examination with blood laboratory tests, electrocardiography as well as assessment of general physical and neurological status. Hence, none of the subjects suffered from diabetes or required insulin administration. Pregnancy of female participants was excluded by urine pregnancy tests before the PET/MR scan. Further exclusion criteria were history of or current substance abuse as well as usage of medication. All participants provided written informed consent after detailed explanation of the study protocol. The study was approved by the Ethics Committee of the Medical University of Vienna (ethics nr: 1916/2013) and procedures were carried out according to the Declaration of Helsinki.

### PET/MR data acquisition

**Fig. 1:**
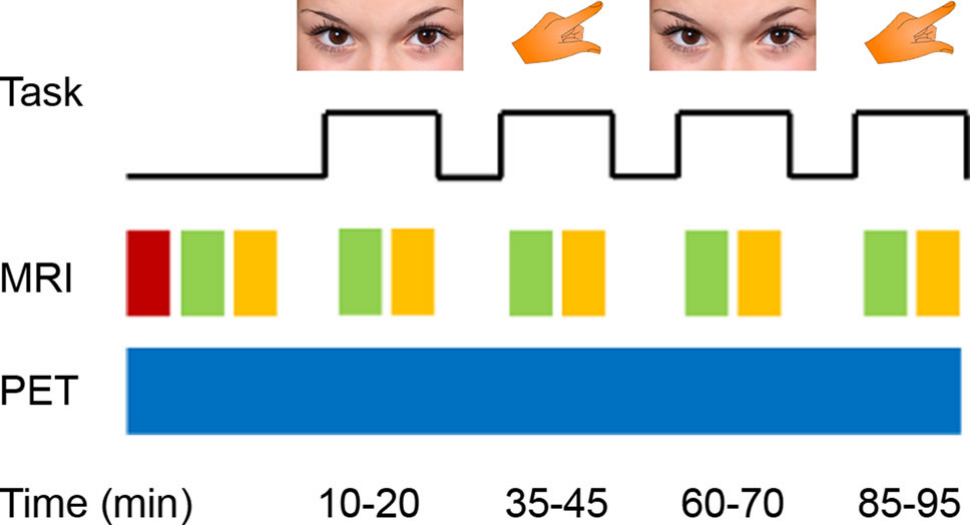
Schematic diagram of the PET/MR acquisition and the task. Subjects opened their eyes (10–20 and 60–70 min) or tapped the right thumb to the fingers (35–45 and 85–95 min), with eyes closed and no finger movement otherwise. At baseline, a T1-weighted structural image (red) was acquired. Functional (green) and diffusion weighted MRI (yellow) was obtained at baseline as well as during the continuous task performance. PET (blue) was acquired simultaneously with the MRI. Images partly taken from pixabay.com under the CC0 creative common license

All subjects underwent one examination with a combined PET/MR system (Siemens mMR, Erlangen, Germany). Participants fasted for at least 5.5 h before radioligand application (Varrone et al. 2009). Head movement was minimized by placing foam pads between the head and the head coil. During the measurement, subjects opened their eyes (10–20 and 60–70 min after [^18^F]FDG infusion start) or repeatedly tapped the right thumb to the fingers (35–45 and 85–95 min, Fig. 1). Otherwise, eyes were closed and no finger movement was carried out. None of the subjects reported to having fallen asleep during the scan.

Recording of 95 min PET data in list-mode started simultaneously with the radioligand administration. [^18^F]FDG (2-[^18^F]fluoro-2-deoxy-d-glucose) was daily prepared according to an established procedure (Hamacher et al. 1986) with the GE FASTlab platform (General Electric Medical Systems, Waukesha, WI, USA). The radioligand was applied as constant infusion during the entire scan (injected dose = 3 MBq/kg body weight, pump speed = 36 ml/h) (Hahn et al. 2016a; Villien et al. 2014) (Volumed μVP7000, Arcomed, Regensdorf, Switzerland). Attenuation correction of PET data was realized with a separate low-dose CT scan (Siemens Biograph TruePoint PET/CT). The CT was spatially coregistered to the T1-weighted image with SPM12 and scaled bi-linearly to obtain an attenuation map (Carney et al. 2006; Ladefoged et al. 2015). Since the CT was not available in one subject, the T1-weighted image was converted to a pseudoCT, which has been shown to represent a robust substitute (Burgos et al. 2014). Although this represent a potential limitation of the study, the CMRGlu values of this subject did not represent outlier values (i.e., within group mean ± 2*SD). PET images were then reconstructed to 95 × 1 min frames, spatial resolution was 4.3 mm full-width at half-maximum 1 cm next to the center of the field of view (voxel size = 2.09 × 2.09 × 2.03 mm).

Simultaneously with the PET acquisition, functional MRI (fMRI) and diffusion weighted imaging (DWI) were recorded at rest before the first task as well as during all four task blocks, i.e., each sequence 2× for the eyes open condition and 2× for right finger tapping. For fMRI, an echo-planar imaging sequence was used (TE/TR = 30/2440 ms, 5 min per acquisition, voxel size = 2.1 × 2.1 × 3 mm + 0.75 mm slice gap). Diffusion weighted images were acquired with an echo-planar imaging sequence in 30 diffusion-encoding directions and two non-diffusion weighted images (TE/TR = 76/8000 ms, *b* value = 800 s/mm^2^, 5.5 min per acquisition, voxel size = 2 × 2 × 2 mm). Furthermore, a T1-weighted structural image was acquired at rest before start of the PET acquisition for spatial registration purposes (magnetization prepared rapid gradient echo sequence, TE/TR = 4.2/2000 ms, voxel size = 1 × 1 × 1.1 mm).

### Blood sampling

Right before start of the PET/MR scan glucose concentration in blood was measured (5.29 ± 0.62 mmol/l). During the scan manual arterial blood samples were taken from the radial artery at 10, 20, 35, 45, 60, 70, 85 and 95 min after infusion start (Hahn et al. 2016a). To avoid interference of the field homogeneity, blood sampling was carried out during MR scanning breaks. For each sample whole blood activities as well as plasma activities (following centrifugation at 2500*g* for 5 min at room temperature) were measured in a gamma counter (Wizard^2^ 2480, Perkin Elmer, Waltham, MA, USA). The arterial input function was then fitted with the sum of two exponentials (Hahn et al. 2016a).

### Data processing

T1-weighted, fMRI and PET data were preprocessed in SPM12 with default parameters unless specified otherwise (Hahn et al. 2016a, b). T1-weighted images were spatially normalized to MNI-space (Montreal Neurological Institute). fMRI data were corrected for slice timing effects (reference = middle slice) and head motion (quality = 1, registered to mean) and spatially normalized to MNI-space. Since SPM12’s spatial normalization is optimized for structural and functional MRI data, PET images were registered to MNI-space via the T1-weighted image as follows. PET data were corrected for head motion (quality = 1, registered to mean) and the mean image was coregistered to the T1-weighted image. The transformation matrices of coregistration (PET mean → T1) and normalization (T1) were then applied to the dynamic PET frames. Normalized fMRI and PET data were finally smoothed with an 8 mm Gaussian kernel.

### Quantification of glucose metabolism

The cerebral metabolic rate of glucose (CMRGlu) at rest and for the two tasks was quantified in two steps as described previously (Hahn et al. 2016a). Briefly, PET images were masked with SPM12’s gray matter tissue prior to exclude non-gray matter voxels and a low-pass filter was applied to reduce artifacts (12th order FIR, cutoff frequency = 5 min). A general linear model (GLM) implemented in Matlab R2011a (The Mathworks, Natick, MA, USA) was then applied to fit voxel-wise time activity curves. Four regressors were included in the GLM, namely one for baseline metabolism (average time course across all gray matter voxels modeled using linear regression with a third order polynomial and task effects included as nuisance variables), one for eyes open and right finger tapping (slope = 1 during the task, slope = 0 otherwise) and one to account for movement-related artifacts (first principal component score from the six realignment parameters of the PET scan). As shown previously (Hahn et al. 2016a; Villien et al. 2014), task-related changes in glucose metabolism are proportionally reflected as changes in the slope of the TAC, which were in turn estimated by the GLM analysis. Hence, in the second step, a Patlak plot was used for the quantification of baseline and task-specific influx constants *K*_i_. Finally, CMRGlu was calculated from *K*_i_ using blood glucose levels and the lumped constant of 0.89.

### Functional connectivity

It has recently been demonstrated that the functional connectivity extracted from conventional fMRI experiments using block designs does not match that of continuous acquisition (Ganger et al. 2015). That is, any design with externally paced stimuli (e.g., block or event related fMRI design) includes fundamentally different signal characteristics due to high-frequency changes at task on- and offsets. Therefore, tasks were carried out continuously for 10 min without external pacing (except for start and end) and fMRI was acquired during this period (Shirer et al. 2012). Functional connectivity was computed for the resting condition as well as each block of eyes open and right finger tapping with Matlab as described previously (Hahn et al. 2015, 2016b). Linear regression was applied to remove potentially confounding signals from white matter and cerebrospinal fluid as well as the six realignment parameters. Global signal was not included in the regression due to recent concerns regarding interpretability (Saad et al. 2012). Afterwards, data were band-pass filtered (12th order FIR filter, 0.007 < *f* < 0.08 Hz). Functional connectivity was calculated by cross-correlating the average time course of a seed region with the entire brain, followed by *z*-transformation. Seeds were defined as brain regions showing significant changes in CMRGlu during the tasks (i.e., primary visual and left motor cortices for eyes open and finger tapping, respectively, all *p* < 0.05 FWE-corrected voxel level). For the conjunction of the two tasks, the seed in the ventromedial prefrontal cortex was defined as intersection of the significant effects in both tasks.

### Tractography and white matter microstructure

DWI data were processed in FSL v5.0.5 with default parameters unless specified otherwise as described previously (Hahn et al. 2016b). After adjustment for eddy currents and head movement, the skull and non-brain tissue was removed using the brain extraction tool. The tensor model was fitted with a weighted least squares approach resulting in maps of axial (L1) and radial diffusivity (RD) as well as fractional anisotropy (FA). L1 and RD maps from the task conditions were coregistered to the baseline scan. Tractography was done in individual space with the diffusion toolbox (Behrens et al. 2003) with 5000 samples streamlines and two fiber directions per voxel to enable tracking of crossing fibers (Behrens et al. 2007). Probabilistic tractography was carried out for the baseline scan between brain regions showing task-relevant changes in CMRGlu (i.e., V1, left M1 and vmPFC) and functional connectivity (i.e., SMA, right M1, thalamus, midbrain, angular gyrus, middle/inferior temporal cortex, posterior insula and the middle cingulate cortex, all *p* < 0.05 FWE-corrected cluster level). These functionally defined brain regions were smoothed and dilated to provide optimal seed regions for tractography. Additional waypoint or exclusion masks were used as appropriate and included the midsagittal line as well as the genu and the body of the corpus callosum. Seed regions and masks were registered to individual space using the inverse transformation matrices of the spatial normalization (T1-weighted images) and the coregistration (FA to T1). To exclude spurious tracts, the lowest 10% of sample streamlines and voxels with FA < 0.2 was removed. To avoid bias regarding the definition of seed and target regions, tracts were modeled in both “directions” (A → B and B → A) and then combined. Finally, mean values of L1 and RD were extracted across the entire tract for rest and task conditions. Averaging of diffusion metrics across an entire tract increases signal-to-noise ratio and is thus less susceptible to spurious findings (Mandl et al. 2013). For visualization purposes but not for quantitative assessment, individual tracts were also registered to MNI-space and averaged across subjects.

### Statistical analysis

All voxel-wise comparisons were corrected for multiple comparisons at *p* < 0.05 FWE-corrected cluster level following *p* < 0.001 uncorrected voxel level. However, seed regions for functional connectivity analysis as obtained from CMRGlu analysis were defined at *p* < 0.05 FWE-corrected voxel level. Significant task-related changes in CMRGlu were assessed by one sample t tests in SPM12 (Hahn et al. 2016a). Task-specific functional connectivity was computed by repeated measures ANOVA in SPM12. All *z*-score functional connectivity maps of the respective task blocks were averaged and compared to the baseline condition (i.e., two functional connectivity task maps for eyes open and right finger tapping; four task maps for the conjunction of the two tasks). Task-specific changes in axial and radial diffusivity were assessed by rmANOVA for each seed region (i.e., primary visual, primary motor and ventromedial prefrontal cortices). Diffusion metrics obtained from repeated task blocks were averaged and compared to the baseline condition.

## Results

**Fig. 2:**
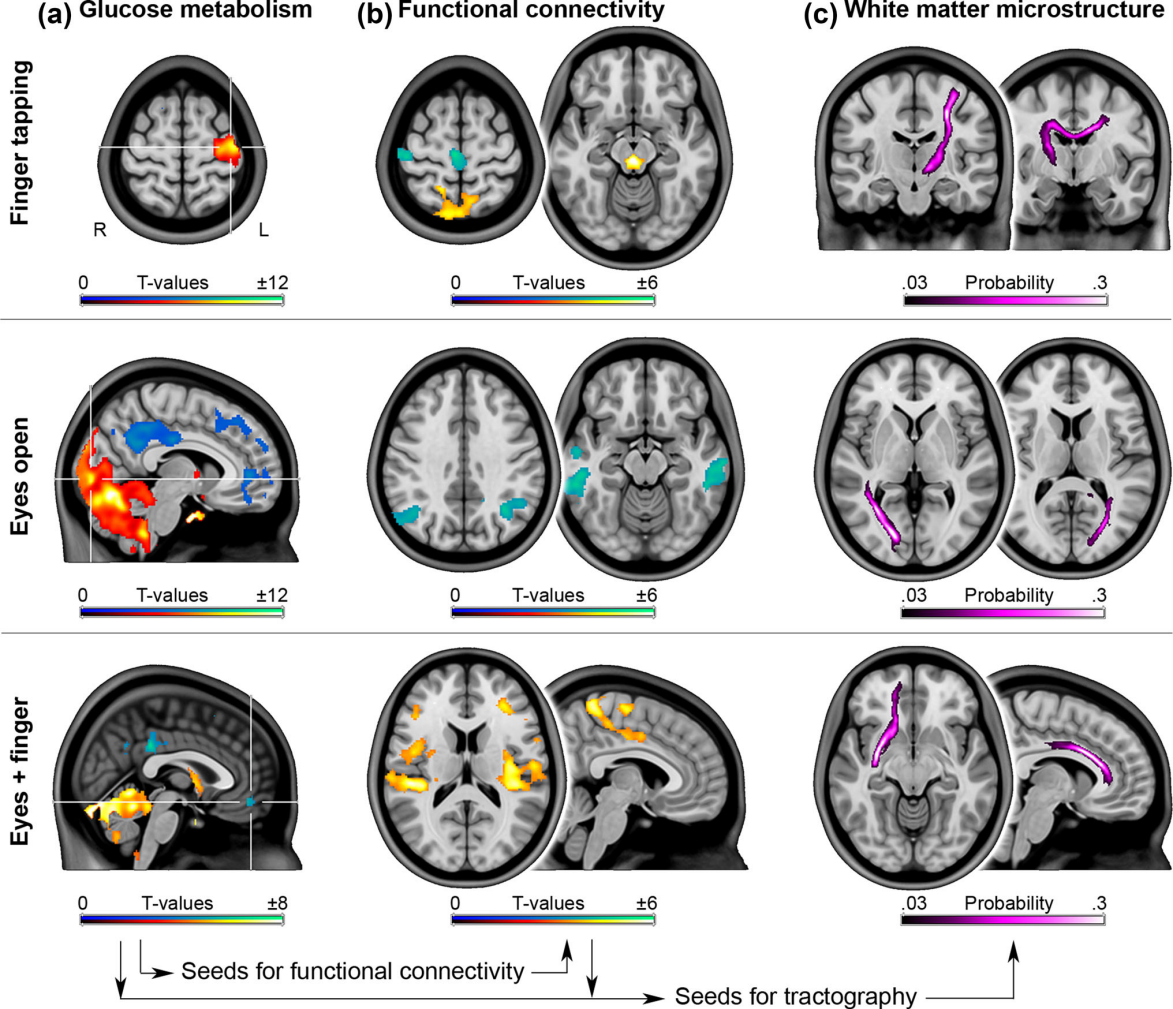
Task-specific changes in glucose metabolism, functional connectivity and the corresponding white matter fiber tracts. **a** Significant changes in glucose metabolism during task performance served as seed regions for functional connectivity (*p* < 0.05 FWE-corrected, seeds marked by crosshair). Common changes of both conditions were assessed by conjunction analysis. **b** Compared to rest, widespread changes in functional connectivity were observed beyond the primary resting-state networks (*p* < 0.05 FWE-corrected). **c** White matter fiber tracts as modeled by probabilistic tractography exhibited task-related changes in radial and axial diffusivity during finger tapping and when eyes were opened, respectively

**Fig. 3:**
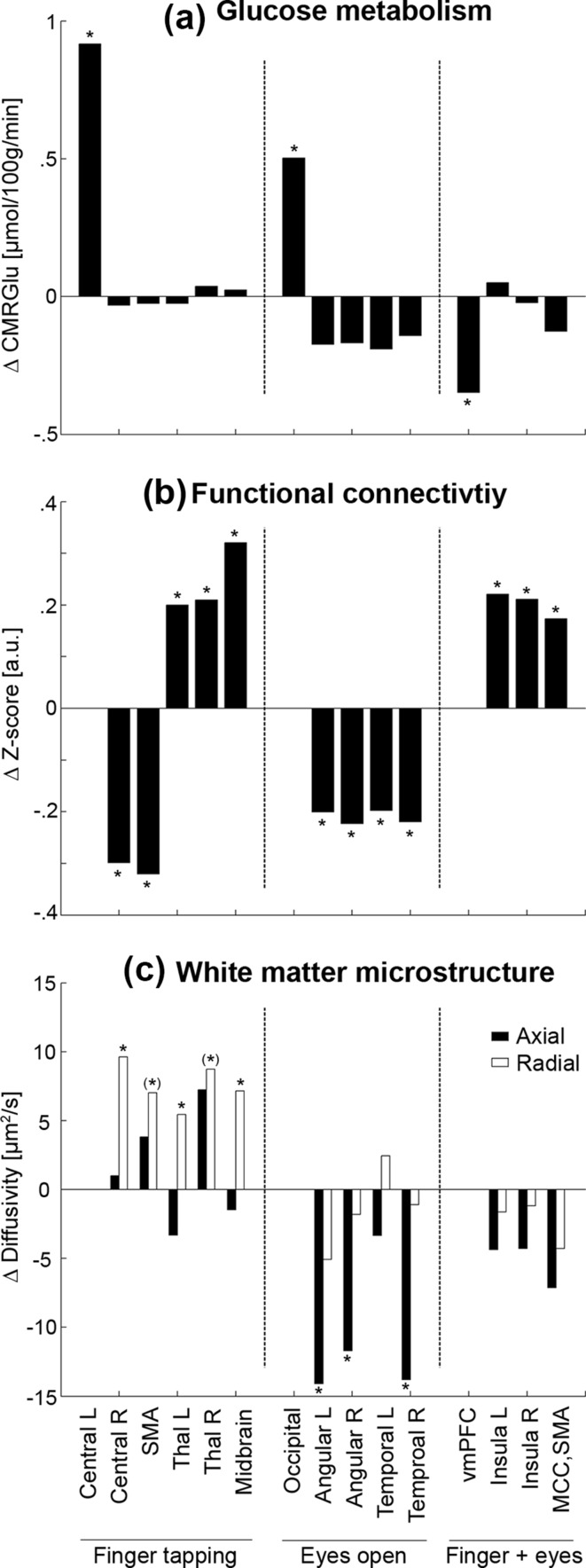
Average changes during task performance as compared to rest for **a** glucose metabolism (µmol/100 g/min), **b** functional connectivity (*z*-score) as well as **c** axial (black) and radial diffusivity (white, µm^2^/s). For **b** and **c** brain regions indicate the connection with the corresponding seed region for each task (central left, occipital cortex and vmPFC). Asterisks denote significant task-related changes at *p < 0.05 or (*) *p* < 0.06

During right finger tapping, increased CMRGlu was observed in the left primary motor cortex (M1) and the cerebellum (peak ΔCMRGlu = 1.0 ± 0.7 µmol/100 g/min = 4.8 ± 2.1%, *p* < 0.05 FWE-corrected, Figs. 2a, 3a, Fig. S1). Using the left M1 as seed region task-specific changes in functional connectivity were observed in various other regions involved in motor function. This included decreases in connectivity during finger tapping with the supplementary motor area (SMA) and the contralateral M1, but increases with the bilateral thalamus and the midbrain (|Δ*z*-score| = 0.27 ± 0.19, *p* < 0.05 FWE-corrected, Figs. 2b, 3b, Fig. S1). Reconstruction of the corresponding white matter fiber pathways depicted direct anatomical connections between these regions such as the corticospinal tract and contralateral projections passing through the body of the corpus callosum (Fig. 2c). Importantly, radial diffusivity consistently increased across all left M1 tracts during finger tapping by around 1.5 ± 2.7% (*F*_1,17.3_ = 9.2, *p* = 0.007, Fig. 3c).

Opening the eyes resulted in increased CMRGlu in the primary visual cortex (V1) and the cerebellum, as compared to eyes closed (ΔCMRGlu = 0.5 ± 0.3 µmol/100 g/min = 1.7 ± 0.6%, *p* < 0.05 FWE-corrected, Figs. 2a, 3a). This was accompanied by decreased V1 functional connectivity with the bilateral angular gyri and the middle/inferior temporal cortices (Δ*z*-score = − 0.24 ± 0.20, *p* < 0.05 FWE-corrected, Figs. 2b, 3b), which are part of the dorsal and ventral visual pathways, respectively (Lehky and Sereno 2007). In addition, the reconstructed V1 white matter fiber tracts (Fig. 2c) showed decreased axial diffusivity during the task by − 0.8 ± 1.6% (*F*_1,19.7_ = 10.1, *p* = 0.005), with significant post hoc differences in all but one tract (Fig. 3c).

Both tasks decreased CMRGlu in regions of the frontal cortex and the default mode network (DMN), including the posterior cingulate and the ventromedial prefrontal cortices (vmPFC, ΔCMRGlu = − 0.4 ± 0.3 µmol/100 g/min = − 1.5 ± 1.1%, Fig. 2a, *p* < 0.05 FWE-corrected). This is highly consistent with previous findings using fMRI (Fox and Raichle 2007), but in contrast the approach enabled absolute quantification of such task-related decreases in the DMN. During both tasks functional connectivity of the vmPFC increased with the posterior insula bilaterally and the middle cingulate cortex/SMA (Δ*z*-score = 0.20 ± 0.11, *p* < 0.05 FWE-corrected, Fig. 2b), regions involved in interoception and environmental monitoring, respectively (Taylor et al. 2009). Reconstructed fiber tracts (Fig. 2c) did not show significant differences in diffusivity during the tasks (Fig. 3c).

No significant correlations between imaging parameters were observed.

## Discussion

These results demonstrate that even simple tasks are accompanied by widespread changes across entire brain networks. Except for the SMA and contralateral M1, none of the regions identified with functional connectivity during the tasks were part of their primary resting-state networks (Smith et al. 2009). Also, none of these regions showed relevant changes in CMRGlu (Fig. 3a), indicating that functional connectivity and glucose metabolism may change independently and hence represent complementary information (Wehrl et al. 2013), which cannot be captured by one imaging modality alone. Still, the sensitivity of fPET remains to be investigated. It might be possible that with improved study designs further brain regions might be detected as active, however, the regions identified here match those observed with fMRI (Witt et al. 2008).

Although it has already been demonstrated that such simple task performance affects widespread BOLD signal changes in the brain, this could only be achieved by an unconventionally high signal-to-noise ratio derived from 100 runs (Gonzalez-Castillo et al. 2012). In contrast, we observed widespread changes with a commonly applied design, and importantly, across various imaging modalities. Of note, the task-specific changes were not correlated across the different imaging modalities. This is, however, not surprising since previous studies have shown rather low effect sizes of *r* ~ 0.3 (Tomasi et al. 2013; Nugent et al. 2015), which would require a much larger sample size to detect. On the other hand, the lack of correlation underlines the complimentary information of the different imaging modalities, as similarly argued for other imaging parameters (Aso et al. 2013). Such independent changes may be explained by aerobic glycolysis, where glucose metabolism occurs in excess of oxygen supply (Fox et al. 1988; Vaishnavi et al. 2010). The relevance of aerobic glycolysis has been further extended to various pathologies such as Alzheimer’s disease (Vlassenko et al. 2010) and high-grade brain tumors (Vlassenko et al. 2015). Hence, the complementary information provided by the different imaging modalities might offer novel tools to investigate coupled changes during task performance as well as pathological alterations thereof.

The combined information of CMRGlu and functional connectivity enabled the identification of specific regions which are subject to accompanying task-related changes as well as the amplitude and direction (increased or decreased connectivity) of these changes. Interestingly, differences in functional connectivity when switching between rest and task conditions were numerically markedly similar (Δ*z* ~ 0.24), independent of the connection, the task as well as decreases or increases in connectivity. These changes in functional connectivity may reflect the level of recruitment of certain brain regions for each task. For instance, angular and temporal gyri represent higher order visual areas (Lehky and Sereno 2007), which are unlikely to be involved when simply opening the eyes and thus exhibited decreased functional connectivity with V1. Similarly, SMA and contralateral M1 are regions involved in bimanual motor coordination (Gerloff and Andres 2002), which is not required in this subtle task. On the other hand, we observed increased connectivity during finger tapping with the thalamus and midbrain regions, both involved in motor control. Although these motor areas would also have been identified as activated using fMRI (Witt et al. 2008), the task-specific functional connectivity draws a much more complex picture, such as changes in M1 connectivity in both directions. This characterization of increased and decreased functional connectivity when switching between rest and task may provide novel information to extend a recently introduced approach of modeling interregional communication and signaling hierarchies (Riedl et al. 2016).

Stronger connectivity was also observed for vmPFC connectivity with the bilateral posterior insula during both tasks. Increased connectivity between DMN and insula has likewise been reported for wakefulness vs deep sleep (Horovitz et al. 2009), which indicates that this may simply reflect the increased attention and body awareness (Taylor et al. 2009) of active states. In line, the insula has been suggested to represent a hub, switching between DMN and salience network (Menon and Uddin 2010; Craig 2011), with the interaction of posterior and anterior insula processing autonomic response to salient stimuli (Menon and Uddin 2010). The relevance of this regional interplay is further emphasized by frequency alterations in the salience network and anterior DMN in patients with chronic pain (Otti et al. 2013) and the corresponding (de)activations during pain processing in healthy individuals (Kucyi et al. 2013). Generally, deactivation of the DMN is a well-described characteristic of task performance (Shulman et al. 1997). Importantly, altered deactivation has been reported in mental disorders such as Alzheimer’s dementia (Lustig et al. 2003) and major depression (Sheline et al. 2009), whereas for the latter this also seems to be an important metric for prediction of early treatment response (Spies et al. 2016). The employed approach to assess glucose metabolism dynamically during tasks now enables absolute quantification of such deactivations and may therefore aid to establish robust markers for diagnosis and treatment prediction. Of note, decreases in glucose metabolism as compared to baseline are reflected in a decreased slope of the [^18^F]FDG time activity curve as shown in our previous work (Hahn et al. 2016a). Considering that blood flow independently changed by more than 100% during hypercapnia without affecting [^18^F]FDG uptake (Villien et al. 2014), it seems unlikely that blood flow considerably influences the PET signal. Similarly, PET time activity curves of dopamine radioligands were not affected by changes in blood flow (Sander et al. 2017).

Regarding task-related changes in white matter microstructure, the exact mechanisms are still unknown. Though, osmotic cell swelling during neuronal activation has been identified as a major underlying factor (Darquie et al. 2001), resulting in decreased radial diffusivity of white matter in mice during visual stimulation (Spees et al. 2013) and humans during acute nicotine challenge (Kochunov et al. 2013). Alternatively, a mathematical model proposed an increase in radial diffusivity due to increased water diffusion through open ion channels (Makris et al. 2014). Since the assessment of task-induced changes in white matter is a rather novel field, further work is required to determine the underlying causes of the observed differences in diffusion metrics. Specifically, it needs to be clarified if the changes during task performance generally show topological (i.e., tract-specific) differences or if these are stimulus dependent, hence, if they can be explained by the nature of a certain task such as the sensory/perceptional constant tasks (eyes open) vs the motoric/executive tasks with high pacing (finger tapping) used here. In support of the former hypothesis, independent work observed markedly similar results as ours, namely task-related changes in radial and axial diffusivity during finger and visual stimulation, respectively (Mandl et al. 2013). Although we were able to detect task-related changes in white matter microstructure, the optimal setting still needs to be established. This is especially true for the acquisition parameters, task duration and/or number of repetitions as well as image processing. Since the expected effects are smaller as compared to, e.g., functional MRI experiments (Aso et al. 2013), averaging across entire tracts (Mandl et al. 2013) and individual tract delineation seem to be required to obtain sufficient signal-to-noise ratio. Finally, the potential influence of task-related changes in blood flow on diffusion metrics needs to be considered. Diffusion MRI may be affected by blood microcirculation. However, with increasing *b* values the vascular effects decrease and diffusion effects dominate (Le Bihan 2012). Furthermore, there was no correlation between task-related diffusion and BOLD signal strengths in gray matter, despite the regionally similar activation, which indicates that these processes are based on different neurophysiological effects (Aso et al. 2013). That is, task-related diffusion MRI has been argued to be more directly related to the neuronal response, whereas BOLD imaging is dependent on the neurovascular coupling (Le Bihan 2012; Aso et al. 2013). Still, the exact influence of blood flow and other vascular effects on task-related changes in white matter diffusion metrics remains to be established.

As another technical issue, the potential influence of PET on the MR acquisition needs to be highlighted. Although the MR part in general does not seem to be affected by PET (Delso et al. 2011), stronger eddy current effects were observed for diffusion imaging in hybrid scanner systems (Boss et al. 2010). This may lead to uncertainties in spatial registration, which is, however, not an issue in the current study since tractography was carried out in individual space. On the other hand, the PET system did not significantly affect principal eigenvector angles or FA maps (Boss et al. 2010). We would like to mention that dedicated stand-alone MR scanners may outperform the integrated MR of the hybrid system. This includes field strengths beyond 3T, gradient strengths and the number of channels included in the head coil (e.g., 80 mT/m and 64 channels in the Siemens Prisma). Still, the used PET/MR is comparable to most stand-alone MR scanners which are used in clinical routine applications.

To summarize, we highlight that even simple task performance yields substantial changes in human brain glucose metabolism, functional connectivity and white matter microstructure. These differences illustrate task-specific contributions of brain regions beyond the primary resting-state networks and demonstrate the complementary nature of the different imaging modalities.

## Electronic supplementary material

Below is the link to the electronic supplementary material.

Supplementary material 1 (DOCX 218 kb)

## References

[CR1] Aso T, Urayama S, Fukuyama H, Le Bihan D (2013). Comparison of diffusion-weighted fMRI and BOLD fMRI responses in a verbal working memory task. Neuroimage.

[CR2] Behrens TE, Woolrich MW, Jenkinson M, Johansen-Berg H, Nunes RG, Clare S, Matthews PM, Brady JM, Smith SM (2003). Characterization and propagation of uncertainty in diffusion-weighted MR imaging. Magn Reson Med.

[CR3] Behrens TE, Berg HJ, Jbabdi S, Rushworth MF, Woolrich MW (2007). Probabilistic diffusion tractography with multiple fibre orientations: what can we gain?. Neuroimage.

[CR4] Biswal BB, Mennes M, Zuo X-N, Gohel S, Kelly C, Smith SM, Beckmann CF, Adelstein JS, Buckner RL, Colcombe S, Dogonowski A-M, Ernst M, Fair D, Hampson M, Hoptman MJ, Hyde JS, Kiviniemi VJ, Kötter R, Li S-J, Lin C-P, Lowe MJ, Mackay C, Madden DJ, Madsen KH, Margulies DS, Mayberg HS, McMahon K, Monk CS, Mostofsky SH, Nagel BJ, Pekar JJ, Peltier SJ, Petersen SE, Riedl V, Rombouts SARB, Rypma B, Schlaggar BL, Schmidt S, Seidler RD, Siegle GJ, Sorg C, Teng G-J, Veijola J, Villringer A, Walter M, Wang L, Weng X-C, Whitfield-Gabrieli S, Williamson P, Windischberger C, Zang Y-F, Zhang H-Y, Castellanos FX, Milham MP (2010). Toward discovery science of human brain function. Proc Natl Acad Sci USA.

[CR5] Boss A, Kolb A, Hofmann M, Bisdas S, Nagele T, Ernemann U, Stegger L, Rossi C, Schlemmer HP, Pfannenberg C, Reimold M, Claussen CD, Pichler BJ, Klose U (2010). Diffusion tensor imaging in a human PET/MR hybrid system. Investig Radiol.

[CR6] Burgos N, Cardoso MJ, Thielemans K, Modat M, Pedemonte S, Dickson J, Barnes A, Ahmed R, Mahoney CJ, Schott JM, Duncan JS, Atkinson D, Arridge SR, Hutton BF, Ourselin S (2014). Attenuation correction synthesis for hybrid PET-MR scanners: application to brain studies. IEEE Trans Med Imaging.

[CR7] Cahill L, Haier RJ, Fallon J, Alkire MT, Tang C, Keator D, Wu J, McGaugh JL (1996). Amygdala activity at encoding correlated with long-term, free recall of emotional information. Proc Natl Acad Sci USA.

[CR8] Carney JP, Townsend DW, Rappoport V, Bendriem B (2006). Method for transforming CT images for attenuation correction in PET/CT imaging. Med Phys.

[CR9] Craig AD (2011). Significance of the insula for the evolution of human awareness of feelings from the body. Ann N Y Acad Sci.

[CR10] Darquie A, Poline JB, Poupon C, Saint-Jalmes H, Le Bihan D (2001). Transient decrease in water diffusion observed in human occipital cortex during visual stimulation. Proc Natl Acad Sci USA.

[CR11] Delso G, Furst S, Jakoby B, Ladebeck R, Ganter C, Nekolla SG, Schwaiger M, Ziegler SI (2011). Performance measurements of the Siemens mMR integrated whole-body PET/MR scanner. J Nucl Med.

[CR12] Fair DA, Schlaggar BL, Cohen AL, Miezin FM, Dosenbach NU, Wenger KK, Fox MD, Snyder AZ, Raichle ME, Petersen SE (2007). A method for using blocked and event-related fMRI data to study “resting state” functional connectivity. Neuroimage.

[CR13] Fox MD, Raichle ME (2007). Spontaneous fluctuations in brain activity observed with functional magnetic resonance imaging. Nat Rev Neurosci.

[CR14] Fox PT, Raichle ME, Mintun MA, Dence C (1988). Nonoxidative glucose consumption during focal physiologic neural activity. Science.

[CR15] Ganger S, Hahn A, Kublbock M, Kranz GS, Spies M, Vanicek T, Seiger R, Sladky R, Windischberger C, Kasper S, Lanzenberger R (2015). Comparison of continuously acquired resting state and extracted analogues from active tasks. Hum Brain Mapp.

[CR16] Gerloff C, Andres FG (2002). Bimanual coordination and interhemispheric interaction. Acta Psychol (Amst).

[CR17] Giorgio A, Santelli L, Tomassini V, Bosnell R, Smith S, De Stefano N, Johansen-Berg H (2010). Age-related changes in grey and white matter structure throughout adulthood. Neuroimage.

[CR18] Gonzalez-Castillo J, Saad ZS, Handwerker DA, Inati SJ, Brenowitz N, Bandettini PA (2012). Whole-brain, time-locked activation with simple tasks revealed using massive averaging and model-free analysis. Proc Natl Acad Sci USA.

[CR19] Hahn A, Kranz GS, Seidel EM, Sladky R, Kraus C, Kublbock M, Pfabigan DM, Hummer A, Grahl A, Ganger S, Windischberger C, Lamm C, Lanzenberger R (2013). Comparing neural response to painful electrical stimulation with functional MRI at 3 and 7T. Neuroimage.

[CR20] Hahn A, Kranz GS, Sladky R, Ganger S, Windischberger C, Kasper S, Lanzenberger R (2015). Individual diversity of functional brain network economy. Brain Connect.

[CR21] Hahn A, Gryglewski G, Nics L, Hienert M, Rischka L, Vraka C, Sigurdardottir H, Vanicek T, James GM, Seiger R, Kautzky A, Silberbauer L, Wadsak W, Mitterhauser M, Hacker M, Kasper S, Lanzenberger R (2016). Quantification of task-specific glucose metabolism with constant infusion of 18F-FDG. J Nucl Med.

[CR22] Hahn A, Kranz GS, Sladky R, Kaufmann U, Ganger S, Hummer A, Seiger R, Spies M, Vanicek T, Winkler D, Kasper S, Windischberger C, Swaab DF, Lanzenberger R (2016). Testosterone affects language areas of the adult human brain. Hum Brain Mapp.

[CR23] Hamacher K, Coenen HH, Stocklin G (1986). Efficient stereospecific synthesis of no-carrier-added 2-[18F]-fluoro-2-deoxy-d-glucose using aminopolyether supported nucleophilic substitution. J Nucl Med.

[CR24] Honey GD, Fu CH, Kim J, Brammer MJ, Croudace TJ, Suckling J, Pich EM, Williams SC, Bullmore ET (2002). Effects of verbal working memory load on corticocortical connectivity modeled by path analysis of functional magnetic resonance imaging data. Neuroimage.

[CR25] Horovitz SG, Braun AR, Carr WS, Picchioni D, Balkin TJ, Fukunaga M, Duyn JH (2009). Decoupling of the brain’s default mode network during deep sleep. Proc Natl Acad Sci USA.

[CR26] Kochunov P, Du X, Moran LV, Sampath H, Wijtenburg SA, Yang Y, Rowland LM, Stein EA, Hong LE (2013). Acute nicotine administration effects on fractional anisotropy of cerebral white matter and associated attention performance. Front Pharmacol.

[CR27] Kucyi A, Salomons TV, Davis KD (2013). Mind wandering away from pain dynamically engages antinociceptive and default mode brain networks. Proc Natl Acad Sci USA.

[CR28] Ladefoged CN, Benoit D, Law I, Holm S, Kjaer A, Hojgaard L, Hansen AE, Andersen FL (2015). Region specific optimization of continuous linear attenuation coefficients based on UTE (RESOLUTE): application to PET/MR brain imaging. Phys Med Biol.

[CR29] Le Bihan D (2012). Diffusion, confusion and functional MRI. Neuroimage.

[CR30] Lehky SR, Sereno AB (2007). Comparison of shape encoding in primate dorsal and ventral visual pathways. J Neurophysiol.

[CR31] Logothetis NK (2008). What we can do and what we cannot do with fMRI. Nature.

[CR32] Lustig C, Snyder AZ, Bhakta M, O’Brien KC, McAvoy M, Raichle ME, Morris JC, Buckner RL (2003). Functional deactivations: change with age and dementia of the Alzheimer type. Proc Natl Acad Sci USA.

[CR33] Makris N, Gasic GP, Garrido L (2014). The ionic DTI model (iDTI) of dynamic diffusion tensor imaging (dDTI). MethodsX.

[CR34] Mandl RC, Schnack HG, Zwiers MP, Kahn RS, Hulshoff Pol HE (2013). Functional diffusion tensor imaging at 3 Tesla. Front Hum Neurosci.

[CR35] Menon V, Uddin LQ (2010). Saliency, switching, attention and control: a network model of insula function. Brain Struct Funct.

[CR36] Nishizawa S, Kuwabara H, Ueno M, Shimono T, Toyoda H, Konishi J (2001). Double-injection FDG method to measure cerebral glucose metabolism twice in a single procedure. Ann Nucl Med.

[CR37] Nugent AC, Martinez A, D’Alfonso A, Zarate CA, Theodore WH (2015). The relationship between glucose metabolism, resting-state fMRI BOLD signal, and GABAA-binding potential: a preliminary study in healthy subjects and those with temporal lobe epilepsy. J Cereb Blood Flow Metab.

[CR38] Otti A, Guendel H, Wohlschlager A, Zimmer C, Noll-Hussong M (2013). Frequency shifts in the anterior default mode network and the salience network in chronic pain disorder. BMC Psychiatry.

[CR39] Phelps ME, Kuhl DE, Mazziota JC (1981). Metabolic mapping of the brain’s response to visual stimulation: studies in humans. Science.

[CR40] Riedl V, Bienkowska K, Strobel C, Tahmasian M, Grimmer T, Forster S, Friston KJ, Sorg C, Drzezga A (2014). Local activity determines functional connectivity in the resting human brain: a simultaneous FDG-PET/fMRI study. J Neurosci.

[CR41] Riedl V, Utz L, Castrillon G, Grimmer T, Rauschecker JP, Ploner M, Friston KJ, Drzezga A, Sorg C (2016). Metabolic connectivity mapping reveals effective connectivity in the resting human brain. Proc Natl Acad Sci USA.

[CR42] Saad ZS, Gotts SJ, Murphy K, Chen G, Jo HJ, Martin A, Cox RW (2012). Trouble at rest: how correlation patterns and group differences become distorted after global signal regression. Brain Connect.

[CR43] Sagi Y, Tavor I, Hofstetter S, Tzur-Moryosef S, Blumenfeld-Katzir T, Assaf Y (2012). Learning in the fast lane: new insights into neuroplasticity. Neuron.

[CR44] Sander CY, Mandeville JB, Wey HY, Catana C, Hooker JM, Rosen BR (2017). Effects of flow changes on radiotracer binding: simultaneous measurement of neuroreceptor binding and cerebral blood flow modulation. J Cereb Blood Flow Metab.

[CR45] Schmidt ME, Ernst M, Matochik JA, Maisog JM, Pan BS, Zametkin AJ, Potter WZ (1996). Cerebral glucose metabolism during pharmacologic studies: test–retest under placebo conditions. J Nucl Med.

[CR46] Sheline YI, Barch DM, Price JL, Rundle MM, Vaishnavi SN, Snyder AZ, Mintun MA, Wang S, Coalson RS, Raichle ME (2009). The default mode network and self-referential processes in depression. Proc Natl Acad Sci USA.

[CR47] Shirer WR, Ryali S, Rykhlevskaia E, Menon V, Greicius MD (2012). Decoding subject-driven cognitive states with whole-brain connectivity patterns. Cereb Cortex.

[CR48] Shulman GL, Corbetta M, Fiez JA, Buckner RL, Miezin FM, Raichle ME, Petersen SE (1997). Searching for activations that generalize over tasks. Hum Brain Mapp.

[CR49] Sladky R, Höflich A, Atanelov J, Kraus C, Baldinger P, Moser E, Lanzenberger R, Windischberger C (2012). Increased neural habituation in the amygdala and orbitofrontal cortex in social anxiety disorder revealed by fMRI. PLoS One.

[CR50] Smith SM, Fox PT, Miller KL, Glahn DC, Fox PM, Mackay CE, Filippini N, Watkins KE, Toro R, Laird AR, Beckmann CF (2009). Correspondence of the brain’s functional architecture during activation and rest. Proc Natl Acad Sci USA.

[CR51] Spees WM, Lin TH, Song SK (2013). White-matter diffusion fMRI of mouse optic nerve. Neuroimage.

[CR52] Spies M, Kraus C, Geissberger N, Auer B, Klöbl M, Tik M, Stürkat IL, Hahn A, Woletz M, Pfabigan DM, Kasper S, Lamm C, Windischberger C, Lanzenberger R (2016) Default mode network deactivation during emotion processing predicts early antidepressant response. Transl Psychiatry (**In Press**) 10.1038/tp.2016.265 PMC5545730 28117844

[CR53] Stender J, Kupers R, Rodell A, Thibaut A, Chatelle C, Bruno MA, Gejl M, Bernard C, Hustinx R, Laureys S, Gjedde A (2015). Quantitative rates of brain glucose metabolism distinguish minimally conscious from vegetative state patients. J Cereb Blood Flow Metab.

[CR54] Taylor KS, Seminowicz DA, Davis KD (2009). Two systems of resting state connectivity between the insula and cingulate cortex. Hum Brain Mapp.

[CR55] Tomasi D, Wang GJ, Volkow ND (2013). Energetic cost of brain functional connectivity. Proc Natl Acad Sci USA.

[CR56] Vaishnavi SN, Vlassenko AG, Rundle MM, Snyder AZ, Mintun MA, Raichle ME (2010). Regional aerobic glycolysis in the human brain. Proc Natl Acad Sci USA.

[CR57] Varrone A, Asenbaum S, Vander Borght T, Booij J, Nobili F, Nagren K, Darcourt J, Kapucu OL, Tatsch K, Bartenstein P, Van Laere K (2009). EANM procedure guidelines for PET brain imaging using [18F]FDG, version 2. Eur J Nucl Med Mol Imaging.

[CR58] Villien M, Wey HY, Mandeville JB, Catana C, Polimeni JR, Sander CY, Zurcher NR, Chonde DB, Fowler JS, Rosen BR, Hooker JM (2014). Dynamic functional imaging of brain glucose utilization using fPET-FDG. Neuroimage.

[CR59] Vlassenko AG, Vaishnavi SN, Couture L, Sacco D, Shannon BJ, Mach RH, Morris JC, Raichle ME, Mintun MA (2010). Spatial correlation between brain aerobic glycolysis and amyloid-beta (Abeta) deposition. Proc Natl Acad Sci USA.

[CR60] Vlassenko AG, McConathy J, Couture LE, Su Y, Massoumzadeh P, Leeds HS, Chicoine MR, Tran DD, Huang J, Dahiya S, Marcus DS, Fouke SJ, Rich KM, Raichle ME, Benzinger TL (2015). Aerobic glycolysis as a marker of tumor aggressiveness: preliminary data in high grade human brain tumors. Dis Mark.

[CR61] Wehrl HF, Hossain M, Lankes K, Liu CC, Bezrukov I, Martirosian P, Schick F, Reischl G, Pichler BJ (2013). Simultaneous PET-MRI reveals brain function in activated and resting state on metabolic, hemodynamic and multiple temporal scales. Nat Med.

[CR62] Witt ST, Laird AR, Meyerand ME (2008). Functional neuroimaging correlates of finger-tapping task variations: an ALE meta-analysis. Neuroimage.

