# Supplementary material for: Task-relevant brain networks identified with simultaneous PET/MR imaging of metabolism and connectivity
Source: Brain Struct Funct. 2017 Nov 13;223(3):1369–78. doi: 10.1007/s00429-017-1558-0 (PMC5869947; doi:10.1007/s00429-017-1558-0)
Supplement: Supplementary file 1 — Supplementary material 1 (DOCX 218 kb) [file 429_2017_1558_MOESM1_ESM.docx]

**Task-relevant brain networks identified with simultaneous PET/MR imaging of metabolism and connectivity**

Andreas Hahn^1^, Gregor Gryglewski^1^, Lukas Nics^2^, Lucas Rischka^1^, Sebastian Ganger^1^,

Helen Sigurdardottir^1^, Chrysoula Vraka^2^, Leo Silberbauer^1^, Thomas Vanicek^1^,

Alexander Kautzky^1^, Wolfgang Wadsak^2,3^, Markus Mitterhauser^2,4^, Markus Hartenbach^2^, Marcus Hacker^2^, Siegfried Kasper^1^, Rupert Lanzenberger^1*^

**SUPPLEMENTARY MATERIAL**


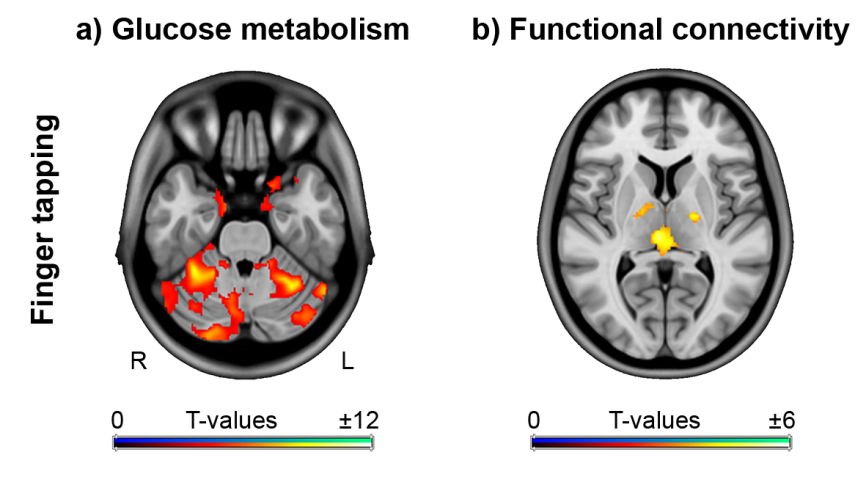


Figure S1: Changes in glucose metabolism and functional connectivity during finger tapping. a) Increases in CMRGlu in the cerebellum were found in the anterior region VI bilaterally, which matches the results of a meta-analysis of fMRI finger tapping tasks (Witt et al. 2008). b) Functional connectivity of M1 increased in thalamic regions which correspond to a connectivity-based parcellation of the human thalamus (Behrens et al. 2003).

**REFERENCES**

Behrens TE, Johansen-Berg H, Woolrich MW, Smith SM, Wheeler-Kingshott CA, Boulby PA, Barker GJ, Sillery EL, Sheehan K, Ciccarelli O, Thompson AJ, Brady JM, Matthews PM (2003) Non-invasive mapping of connections between human thalamus and cortex using diffusion imaging. Nat Neurosci 6 (7):750-757

Witt ST, Laird AR, Meyerand ME (2008) Functional neuroimaging correlates of finger-tapping task variations: an ALE meta-analysis. Neuroimage 42 (1):343-356
